# Supplementary material for: Conformational heterogeneity of molecules physisorbed on a gold surface at room temperature
Source: Nat Commun. 2022 Jul 15;13:4133. doi: 10.1038/s41467-022-31576-x (PMC9287342; doi:10.1038/s41467-022-31576-x)
Supplement: Supplementary file 1 — Supplementary Information [file 41467_2022_31576_MOESM1_ESM.pdf]

Supplementary Information

Conformational heterogeneity of molecules physisorbed on a gold surface at room temperature

Kang et al.

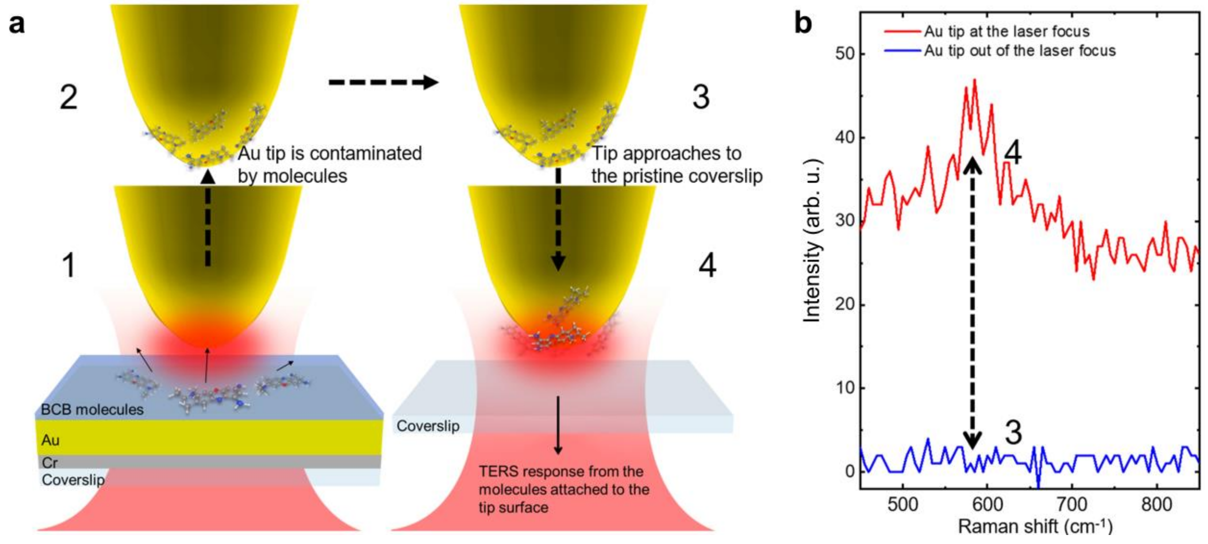

Supplementary Figure 1. Tip contamination process due to the uncapped molecule sample. (a) Schematic illustration describing the tip-contamination procedure by molecule samples in TERS experiments when the molecules are exposed to air without using a thin Al_2O_3 capping layer. During scanning of the Au tip on the molecules, some molecules can cling to the tip surface (procedures 1-2). Hence, plasmon-enhanced Raman response from the molecules sticking on the tip surface can be detected (procedures 3-4). (b) Plasmon-enhanced Raman spectrum of BCB molecules sticking on the tip surface (red). This measurement was performed on the clean surface of a coverslip. When the tip was retracted from the laser focus, the plasmon-enhanced Raman response of BCB molecules was disappeared (blue).

Supplementary Fig. 1a shows a procedure of the tip-contamination problem in TERS experiments when molecule samples are not covered by a thin dielectric capping layer, such as Al_2O_3. The main difficulty of TERS experiments for molecule samples in ambient conditions is rapid spectral diffusions of molecules. I.e., when the strongly confined optical field at the tip apex excites the molecule sample, the thermally excited molecules actively move around and thus robust TERS response is hard to observe. Furthermore, molecules can easily cling to the tip surface during the tip-scanning and the noise TERS response from these molecules spoils TERS experiments. Supplementary Fig. 1b shows a plasmon-enhanced Raman spectrum of BCB molecules sticking on the tip surface (red). This kind of TERS response from the contaminated-tip itself was frequently observed in our TERS experiments when we did not use a thin Al_2O_3 capping layer.

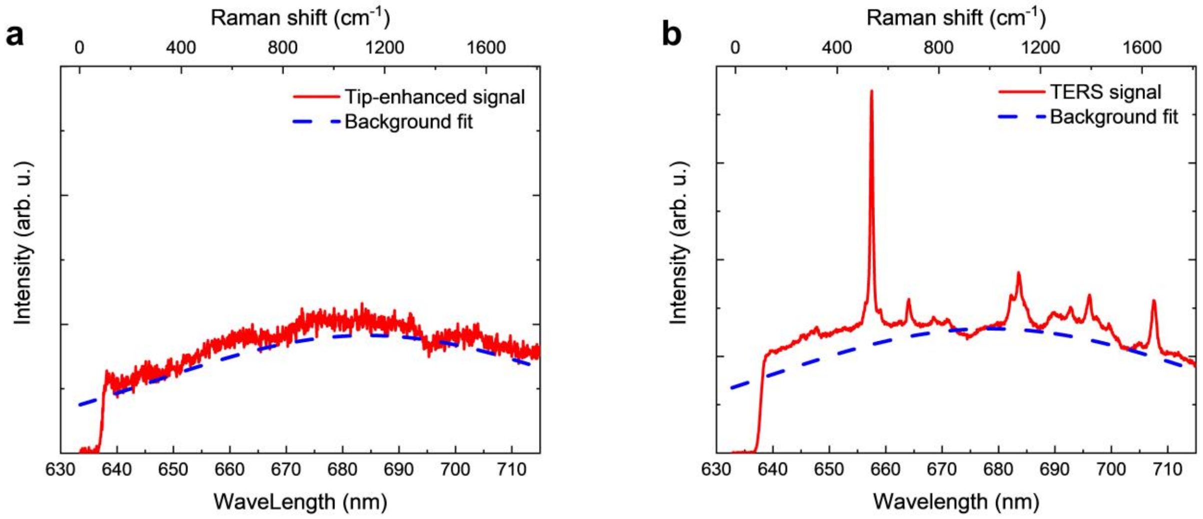

Supplementary Figure 2. Plasmonic background in TERS. Tip-enhanced plasmon response of the Au substrate without BCB molecules (a) and TERS spectrum of BCB molecules on the Au substrate (b). The red solid lines are experimental data and the blue dashed lines are background fit with Voigt function.

Reference	Sample	RMS roughness (nm)
**3**	Thermally evaporated Au layer with molecular adhesives on the coverslip	~0.40
**4**	DC sputtered Au layer with Cu seed layer on silica glass, CaF_2 polished window, and silica oxide	0.20 – 0.30
**5**	Thermally evaporated Au film with Ti adhesion layer (deposition rate of Au: 0.2 Å/s) on Si substrates	~1.13
**5**	Thermally evaporated Au film with Ti adhesion layer (deposition rate of Au: 1.4 Å/s) on Si substrates	~0.50
**Our work**	Thermally evaporated Au film with a thickness of 5 nm with Cr adhesion layer (2 nm) on the coverslip cleaned by piranha solutions (deposition rate of Au: 0.1 Å/s)	~1.90
**Our work**	Thermally evaporated Au film with a thickness of 5 nm with Cr adhesion layer (2 nm) on the coverslip cleaned by sonication and treated by oxygen plasma (deposition rate of Au: 0.1 Å/s)	~1.70
**Our work**	Thermally evaporated Au film with a thickness of 5 nm with Cr adhesion layer (2 nm) on the coverslip cleaned by piranha solutions (deposition rate of Au: 1.0 Å/s)	0.65
**Our work**	Thermally evaporated Au film with a thickness of 5 nm with Cr adhesion layer (2 nm) on the coverslip cleaned by sonication and treated by oxygen plasma (deposition rate of Au: 1.0 Å/s)	0.30 – 0.35

Supplementary Table 1. Comparison of the surface roughness of ultrathin gold films fabricated with different conditions.

The predominant factor determining the surface roughness of the thin Au film is a deposition rate, as described in previous study.[5] When we use a deposition rate of the Au film with 0.1 Å/s, the RMS roughness of metal film was measured to ~1.70 nm and the formation of metallic islands was observed. On the other hand, when we use a deposition rate of the Au film with 1.0 Å/s, the RMS roughness of metal film was measured to ~0.65 nm and the metallic islands were not observed. In addition to the deposition rate, the cleaning method of coverslips also has an effect on the roughness of a thin metal film. When we cleaned coverslips by ultra-sonication with acetone and isopropanol and treated by oxygen plasma, the RMS roughness of the fabricated metal film is decreased two times compared to the metal film using the coverslip cleaned with piranha solutions, as summarized in Supplementary Table 1. By optimizing the deposition rate as well as the cleaning method, we could fabricate flat metal thin films on the coverslip for the bottom-illumination TERS experiment.

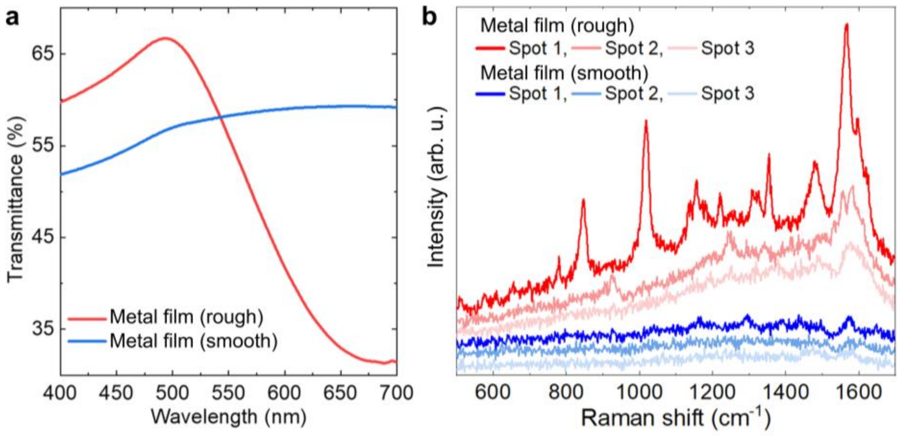

Supplementary Figure 3. Transmittance and spectra of the two different substrate conditions. (a) Measured transmittance spectra of the same samples of Fig. 1c and d. (b) Tip-enhanced optical response spectra of several different spots for the metal films used in Fig. 1c (red) and d (blue). Undesirable tip-enhanced Raman response is observed from the rough metal film (red).

We compare the optical properties of two different metal films in Fig. 1c and d to verify the influence of the surface roughness in TERS experiment. Supplementary Fig. 3a shows transmittance spectra of the metal films measured by UV-Vis spectrophotometer. While the optimized flat metal film shows a uniform transmittance of ~55 % in the broad spectral range (blue), the non-optimal rough metal film shows a resonance characteristic at ~500 nm due to the localized surface plasmon resonance effect of the metal nano-structures (red).[6,7] We then measured tip-enhanced Raman and fluorescence responses of the metal films by approaching the Au tip onto the film surface without BCB molecules. We measure TERS spectra in the three different spots at each metal film with the same Au tip. As shown in supplementary Fig. 3b, on the rough metal film, randomly fluctuating TERS peaks are observed with strong fluorescence signal (red). The fluorescence signals are associated with the plasmon resonance of the formed metal nano-structures, and the random TERS peaks are possibly originated from the physisorbed molecules or contaminants in ambient conditions. By contrast, when we approach the Au tip onto the flat metal film and move the lateral position of it, no significant noise response is observed in most regions (blue). From a large number of measurements, we found that the random TERS peaks vanish significantly for the films that roughness is lower than 1.0 nm.

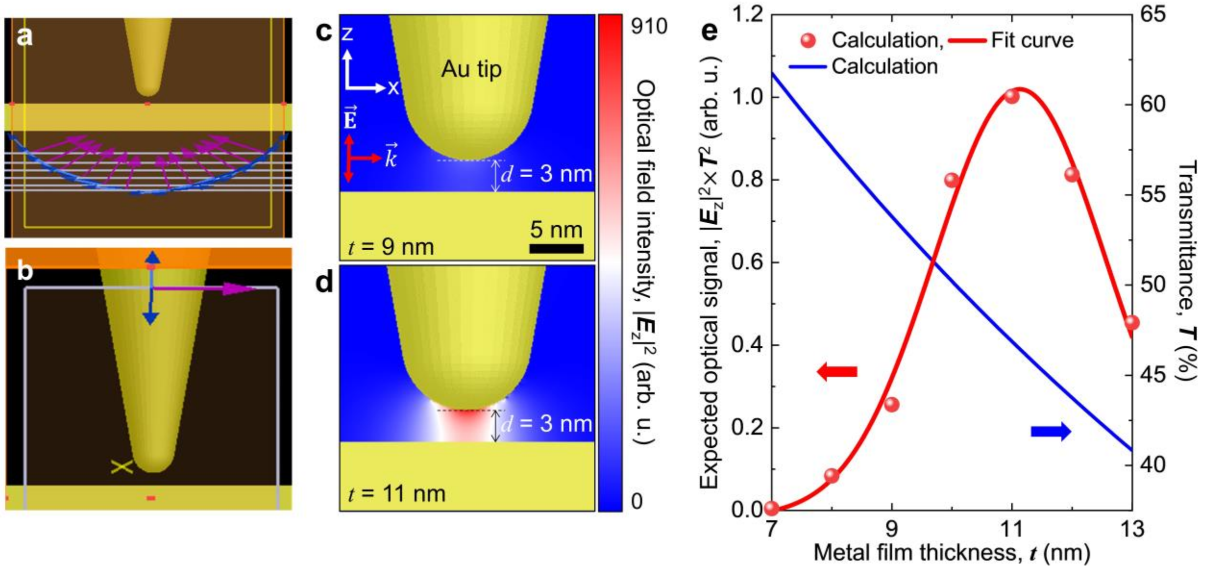

Supplementary Figure 4. Theoretical calculation for different metal film thickness. FDTD simulation conditions for the bottom-illumination geometry with radially polarized light (a) and side-illumination case with linear polarized light (b). (c, d) FDTD-simulated optical field intensity ($|\boldsymbol{E}_z|^2$) distribution at the nano-gap between the Au tip and the thin metal film with the film thickness of 9 nm (c) and 11 nm (d) under the excitation condition of the side-illumination. (e) Expected optical signal (red) in the bottom-illumination mode using the simulation condition of (b) and theoretically calculated transmittance at the wavelength of 632.8 nm (blue) with respect to the thickness of the metal film. The expected optical signal is calculated by the FDTD-simulated optical field intensity at the nano-gap under the simulation condition (b) and multiplied by transmittance $\boldsymbol{T}^2$ at each film thickness as the light passes through the metal film twice. The derived result of the expected optical signal as a function of the metal film thickness is fit with a nonlinear curve (red line).

Supplementary Fig. 4a is the FDTD simulation condition for the Fig. 2b-d. The light sources are arrayed with different polarization conditions by considering the beam focusing by the objective lens (NA = 1.3). In this condition, we obtain the optical field intensity at the tip-sample gap by the excitation source transmitted the Au film likewise the experimental condition. For comparison, we perform the FDTD simulation with a simplified model (supplementary Fig. 4b). In this model, the excitation source directly illuminates the tip-sample gap. Although, the expected optical signal varies more significantly in this model compared to the model of supplementary Fig. 4a, the tendency of the thickness dependent expected optical signal show the same behavior.

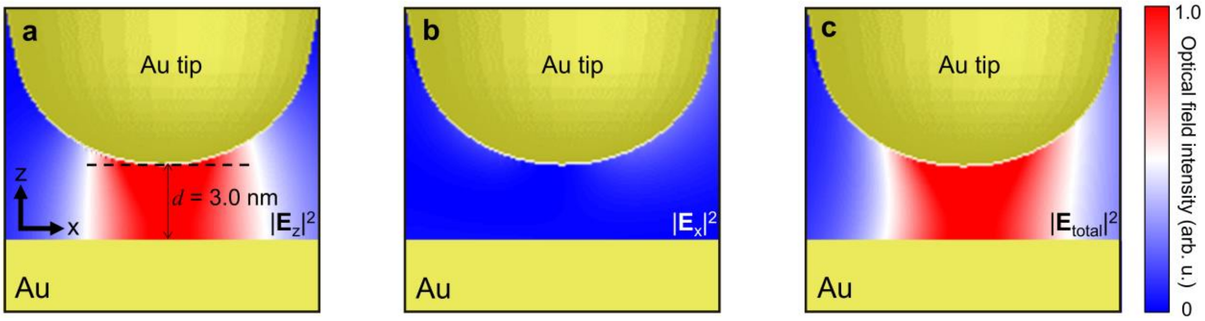

Supplementary Figure 5. FDTD-calculated optical field intensity distributions at the plasmonic nano-gap. FDTD-calculated results of vertical (a) and horizontal (b) field components with the total field intensity (c).

Since the polarization state at the plasmonic nano-gap can affect to the probing vibrational modes of a molecule,[8] we provide the optical field distributions at the nano-gap for the vertical and horizontal field components separately when the excitation light is radially polarized beam,[9] as shown in supplementary Fig. 5. This result clearly shows that the vertical component is dominant at the plasmonic nano-gap and we can selectively probe the out-of-plane vibrational modes in our experiment.

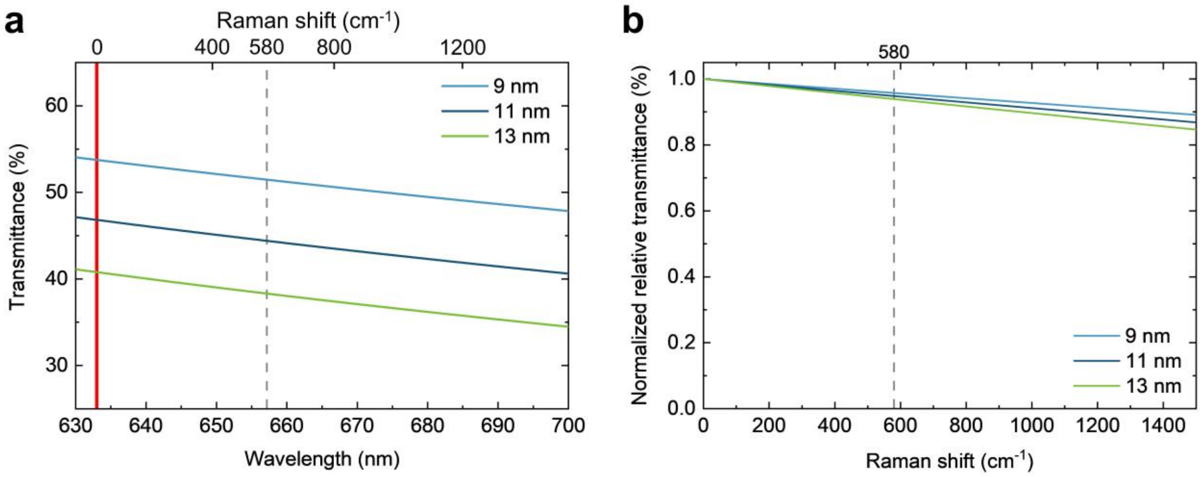

Supplementary Figure 6. Comparison of transmittance of thin Au films with different thicknesses (9, 11, and 13 nm). The absolute value of transmittance of the Au films with respect to the wavelength in the range of 630 nm - 700 nm (a) and the normalized relative transmittance with respect to the Raman shift from 0 cm^{-1} to 1500 cm^{-1} at the wavelength of an excitation light of 633 nm (b).

The transmittance of the metal film is affected by the film thickness as well as the wavelength of light. The transmittance drops almost linearly in the range of 630 nm - 700 nm (supplementary Fig. 6a). When the wavelength of an excitation light is 632.8 nm, the transmittance at 580 cm^{-1} (the strongest Raman mode of BCB molecules) decreases ~5 % compared to the transmittance at 0 cm^{-1} (632.8 nm).

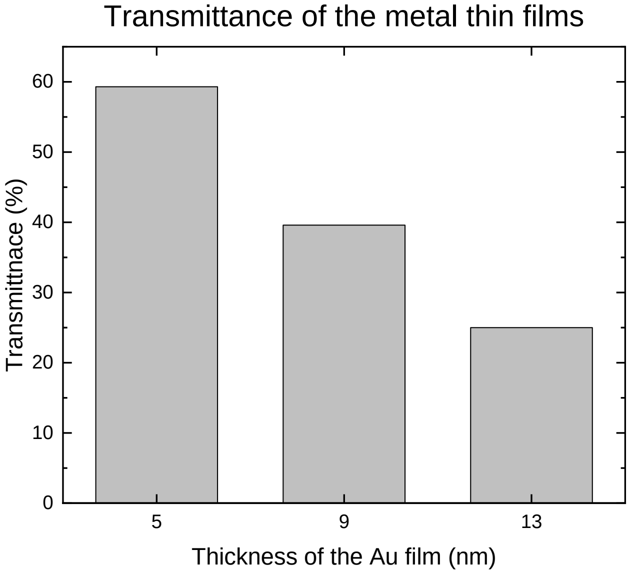

Supplementary Figure 7. The measured transmittance of the metal films with different thickness. The x-axis indicates the thickness of the Au film. Note that these Au thin films have a Cr adhesion layer with a thickness of 2 nm.

The transmittance $\boldsymbol{T}$ of metal thin films with different thickness was measured using an UV-Vis spectrophotometer at 633 nm (an excitation wavelength for TERS experiment). In the bottom-illumination mode TERS experiment, the excitation laser passes through the thin metal film and the TERS signal also passes through the thin metal film. Hence, we need to consider $\boldsymbol{T}^2$ to check the thickness effect of the metal film in the experiment. For example, the $\boldsymbol{T}^2$ value of the Au film with a thickness of 13 nm is about a half compared to the Au film with a thickness of 9 nm.

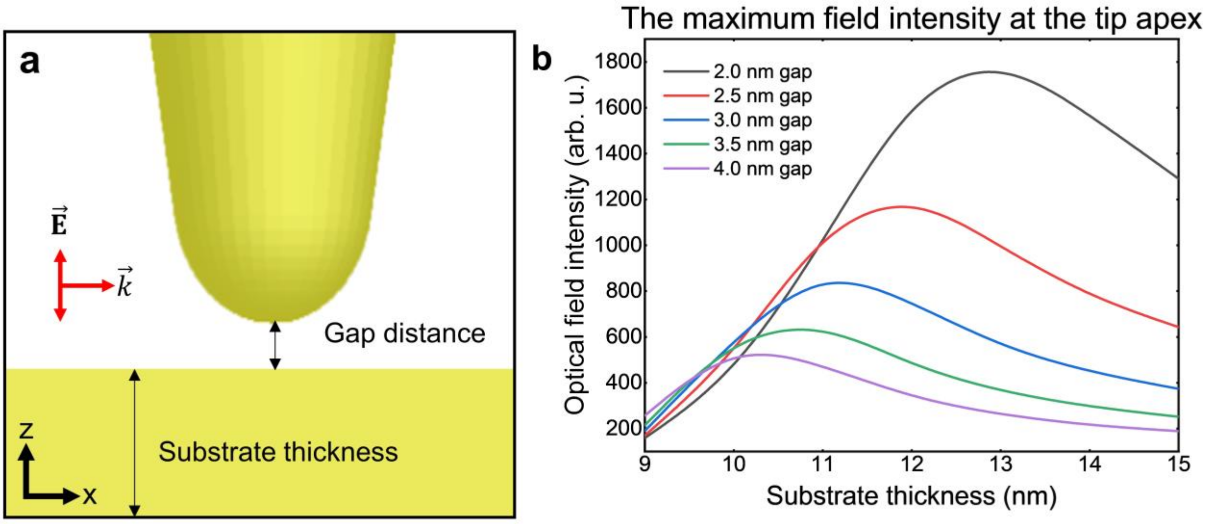

Supplementary Figure 8. FDTD-calculated optical field intensity with varying the metal film thickness and the nano-gap between the Au tip and the Au film. (a) Model for the FDTD simulation indicating the control parameters i.e., gap and thickness. The excitation light with a wavelength of 633 nm is used. (b) FDTD simulation results exhibiting the maximum field intensity at the tip apex with respect to the metal film thickness and the tip-surface gap.

From a series of FDTD simulations, we found that the optical field intensity at the junction between the Au tip and the Au surface is varied depending both on the Au film thickness and the nano-gap. For systematical understanding, we varied the nano-gap distance from 2.0 nm to 4.0 nm with a step of 0.5 nm. At each nano-gap, we varied the metal film thickness from 9 nm to 15 nm, as shown in supplementary Fig. 8b. For the metal films with thickness below 10 nm, the optical field intensity increases with the increasing film thickness, regardless of the nano-gap variation. Interestingly, the maximum field intensity is observed when the film thickness is at around 11 - 13 nm even though the optimal thickness is slightly different depending on the nano-gap. Since the field enhancement is induced by the dipole-dipole interaction between the tip dipole and the image dipole on the metal thin film, we guess that a combination effect of the optimal thickness and the nano-gap gives rise to resonance antenna effect. In general, the nano-gap in a shear-force microscopy is expected to ~3 nm. The simulation result shows that ~11 nm thickness gives maximum field intensity (blue in supplementary Fig. 8b) and this tendency is also confirmed in our experiment. Hence, we believe the optimal metal film thickness for the bottom-illumination mode TERS is about 11 - 12 nm.

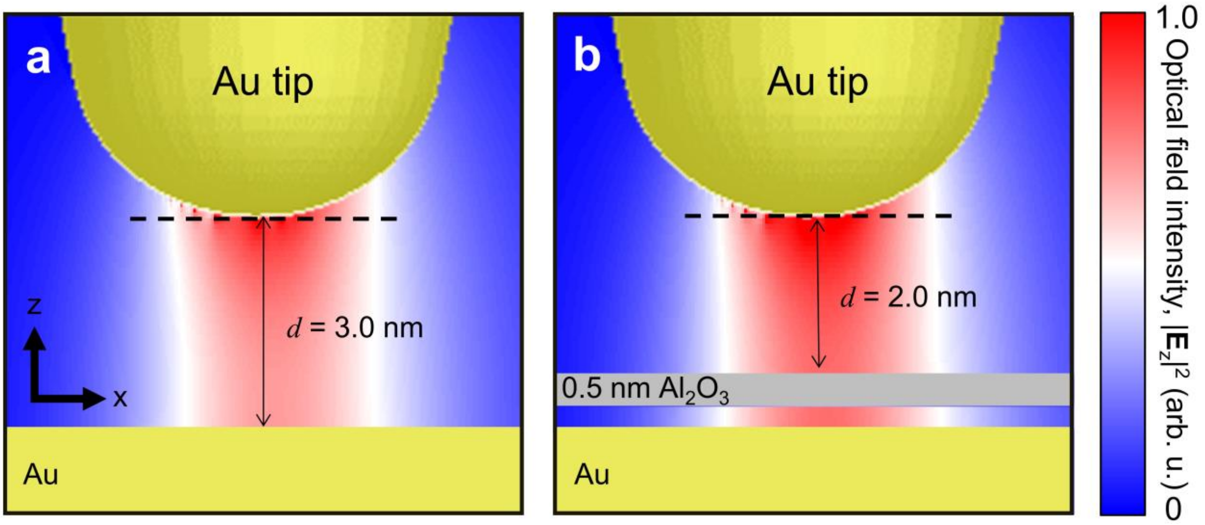

Supplementary Figure 9. FDTD-simulated optical field intensity ($|E_z|^2$) distribution. The field distribution at the nano-gap without (a) and with (b) Al_2O_3 film on Au substrate.

To understand the effect of Al_2O_3 film on the optical field distribution at the plasmonic nano-gap, we performed FDTD simulations with and without the Al_2O_3 film between the Au nano-gap as shown in the supplementary Fig. 9a-b. From these simulations, we confirm that the field distribution at the plasmonic nano-gap is not significantly affected by the existence of Al_2O_3 film.

Supplementary Note 1. Calculation of TERS enhancement factor

We use the following equation for empirical estimation of TERS enhancement for our BCB TERS experiment:[1]

$$\mathrm{EF} = \left(\frac{I_{tip-in} - I_{tip-out}}{I_{tip-out}}\right) \times \frac{A_{FF}}{A_{NF}} \tag{1}$$

where I_{tip-in} is the TERS intensity and $I_{tip-out}$ is the Raman intensity with the tip fully retracted (far-field) from the sample surface. A_{FF} refers to the focused beam spot by an oil-immersion objective lens with NA = 1.30, and A_{NF} refers to near-field excitation region of the Au tip-Au substrate. I_{tip-in} and $I_{tip-out}$ values are derived from the curve fitting by Voigt function, and the values of A_{FF} and A_{NF} derived using a general equation for an area of a circle πr^2. The far-field laser spot radius is $r_{FF} \approx \left(\frac{\lambda}{2NA}\right) \times 1.5 = {\sim}365\, nm$ (the factor 1.5 is the empirical factor)[2] and the near-field radius is $r_{NF} = 10\, nm$, which is a half of the spatial resolution of the Au tip. Using the equation above and our experimental results in Fig. 1b, estimated TERS enhancement factor is as high as ${\sim}2.0 \times 10^5$.

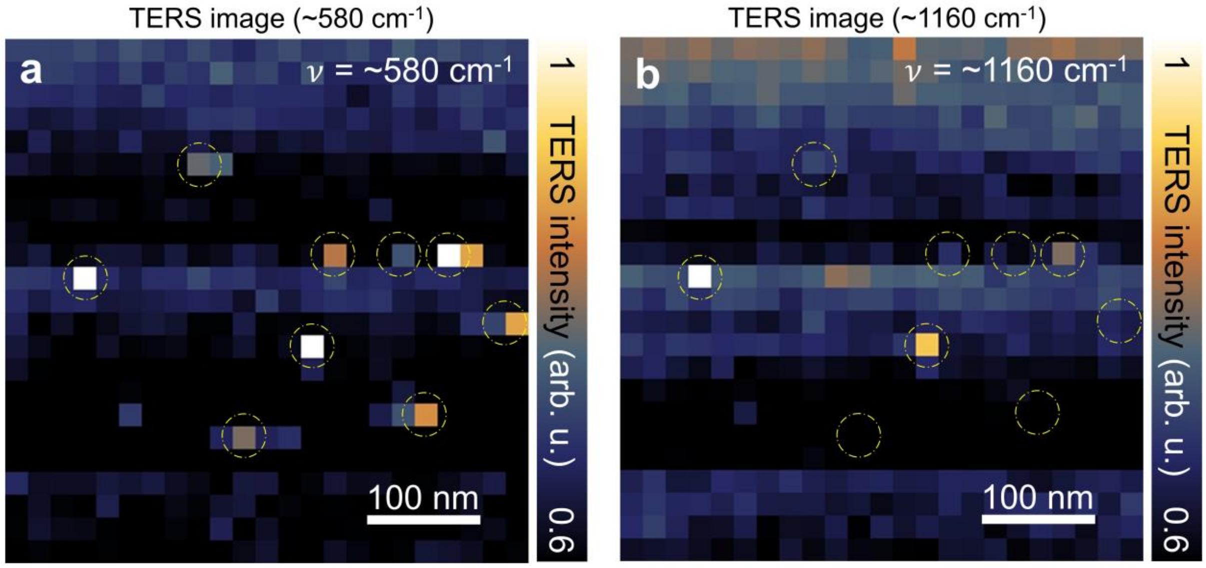

Supplementary Figure 10. Untreated TERS images of single-molecule level TERS. The TERS images for ~580 cm^{-1} (a) and ~1160 cm^{-1} peak intensities (b). These images were postprocessed in Fig. 3a-b of the main text as follows: the line defects along the scanning axis were corrected and an interpolation method was used.

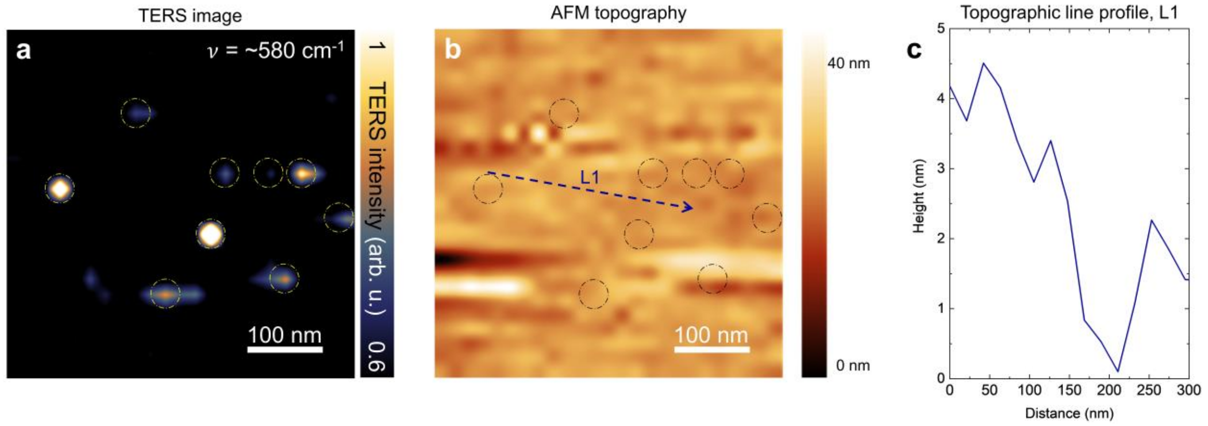

Supplementary Figure 11. TERS image of BCB molecules and corresponding AFM image with a topographic line profile. TERS image for ~580 cm^{-1} vibrational mode (a) and corresponding AFM topography (b). (c) Topographic line profile from the line L1 at (b).

Fig. S11a is a TERS image of BCB molecules for ~580 cm^{-1} vibrational mode and Fig. S11b is the corresponding AFM topography. The indicated 9 circles at the images are the positions of the BCB molecules. In contrast to the TERS image, we cannot distinguish the molecule positions from the topographic image. In our tuning-fork based AFM, the tip-sample distance is stably maintained to ~3 nm, which gives stable TERS signals without perturbation to the intrinsic vibrational modes of molecules. On the other hand, AFM topography is not excellent like STM-TERS due to the drift of both tip scanner and sample scanner caused by thermal expansion at room temperature.

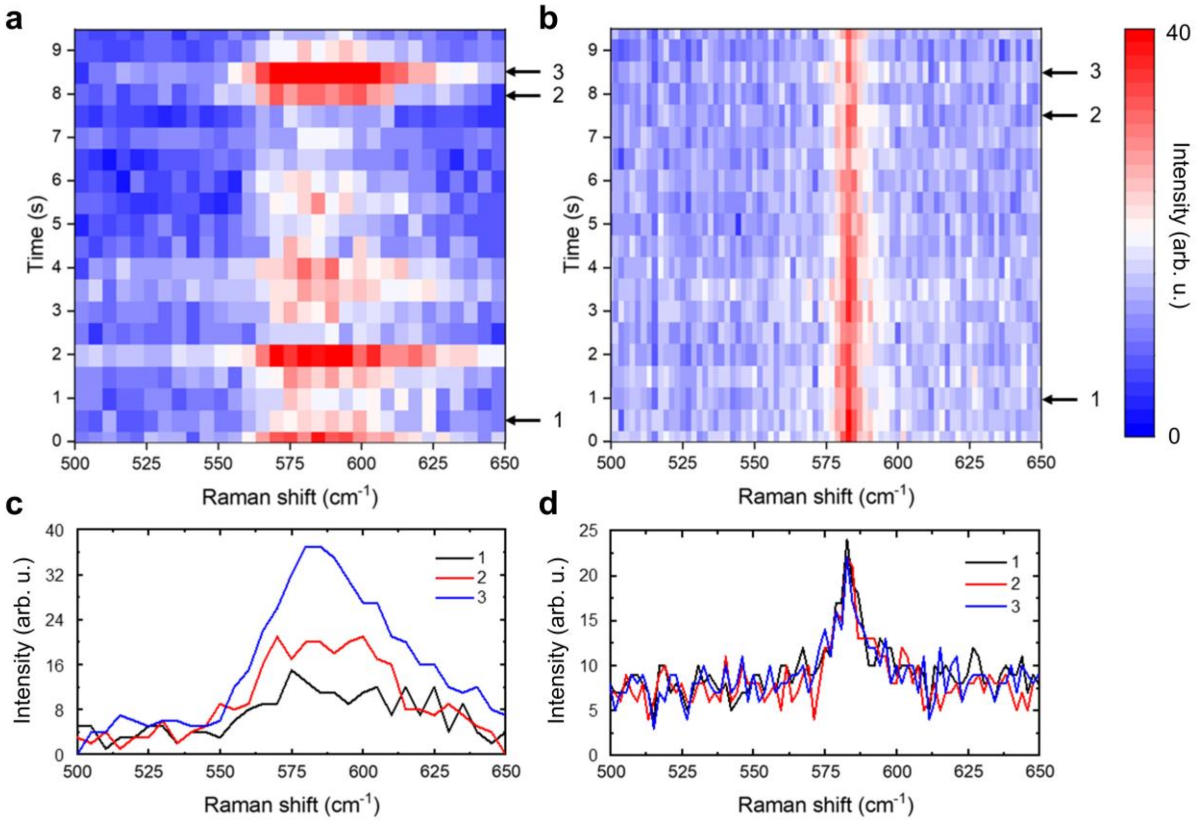

Supplementary Figure 12. Time-series TERS spectra of a few BCB molecules of two different conditions. Time-series TERS spectra without a capping layer (a) and with an Al_2O_3 capping layer (b) under ambient conditions. (c, d) Selected TERS spectra derived from (a) and (b), as indicated by the numbers (1-3).

We performed control experiment to demonstrate the influence of a thin Al_2O_3 capping layer. We measured time-series TERS spectra from a few BCB molecules without the capping layer as shown in Fig. S12a (spectrometer: a grating of 300 grooves/mm with a spectral resolution of ~18.6 cm^{-1}) and with the capping layer as shown in Fig. S12b (spectrometer: a grating of 600 grooves/mm with a spectral resolution of ~4.3 cm^{-1}). From the BCB sample without the capping layer (Fig. S12a and c), we can clearly observe the time-dependent fluctuations in both intensity and peak position due to the rapid spectral diffusions of molecules and their averaging effect in TERS spectra. On the other hand, when we encapsulated the molecules with the capping layer, robust TERS spectra were observed due to the suppressed spectral diffusions of the molecules (Fig. S12b and d). From this result, we verify the significance of a capping layer for molecular TERS experiment in ambient conditions. Note that, in this experiment, all other experimental conditions were controlled, but unfortunately, different gratings were used due to some unavoidable circumstances in the lab.

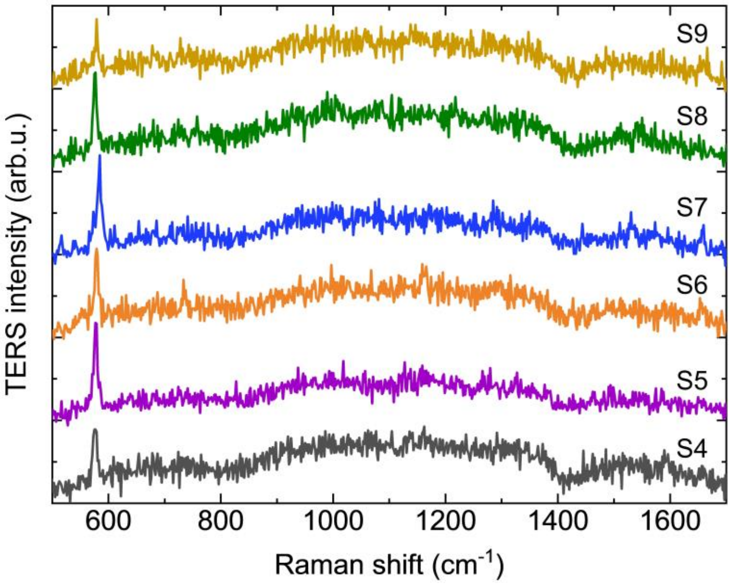

Supplementary Figure 13. TERS spectra measured at the points S4-S9 (Fig. 4a) in the full spectral range (500 cm^{-1} - 1700 cm^{-1}).

	S1	S2	S3	S4	S5	S6	S7	S8	S9
Linwidth (cm^{-1})	6.8	6.6	7.3	7.5	5.4	5.1	6.0	5.6	5.4
Classification	ensemble	ensemble	ensemble	ensemble	single	single	single	single	single

Supplementary Table 2. Comparison of the linewidth of the BCB TERS signals at ~580 cm^{-1} from S1-9 in Fig. 4.

Along with the peak position and intensity, we compare the linewidth of the TERS signals measured at S1-9 for vibrational mode at ~580 cm^{-1} to classify them into the molecular ensembles and single molecules. The group that we classified as ensemble (S1-4) has broader peak linewidth ($\geq$ 6.6 cm^{-1}) than the group of potential single molecules (S5-9 with linewidth of $\leq$ 6.0 cm^{-1}). DFT calculated results show that the BCB molecule has two distinct vibrational modes around 580 cm^{-1}. The peak linewidth shows quite broad even for the single molecules due to the homogeneous broadening caused by the intramolecular coupling at room temperature.

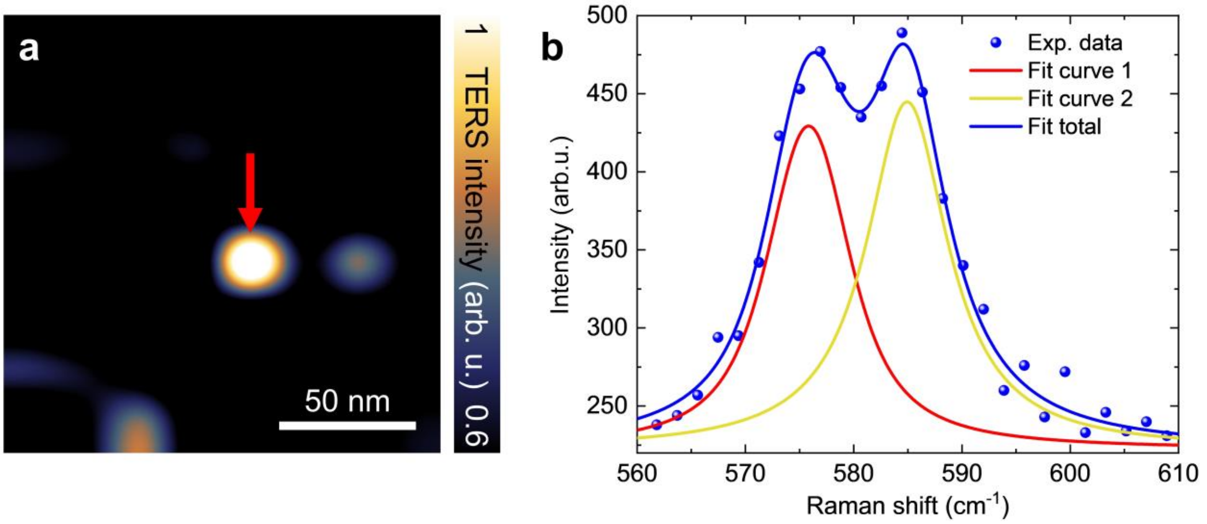

Supplementary Figure 14. TERS image and a TERS spectrum from the image. (a) TERS peak intensity image of BCB molecules for the vibrational mode at ~580 cm^{-1}. (b) TERS spectrum obtained from the red arrow indicated in (a).

Fig. S14a and b show the TERS peak intensity image of BCB molecules for the vibrational mode at ~580 cm^{-1} with TERS spectrum obtained from the red arrow indicated in Fig. S14a. The observed TERS spot is expected to be a few molecules from the consideration of the spatial distribution, peak intensity, and linewidth of the TERS spectrum. From the simple curve fits of the TERS spectrum in Fig. S14b, we can clearly confirm the conformational heterogeneity of a few molecules.

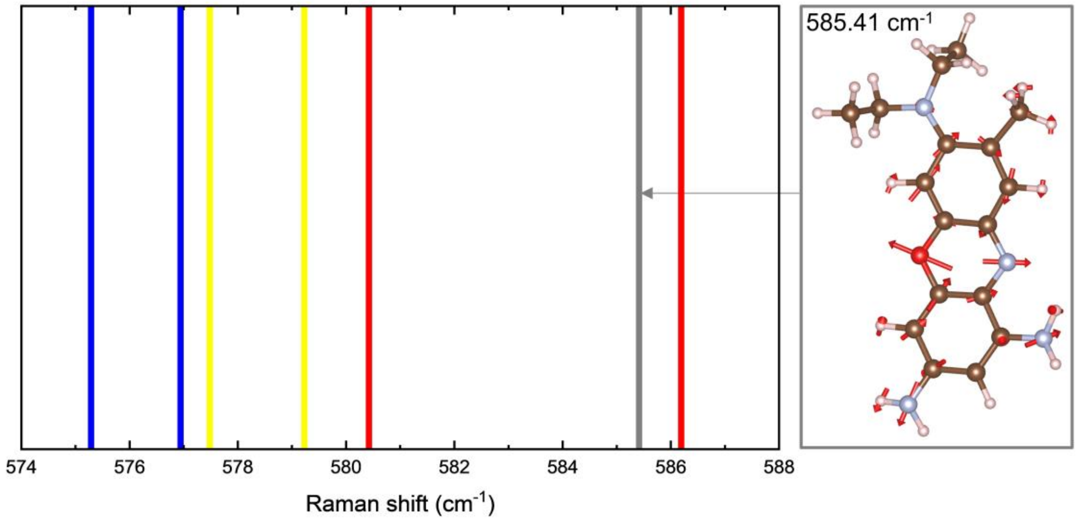

Supplementary Figure 15. DFT calculated vibrational modes of a BCB molecule under different chemical environments. The frequency range is 574 – 588 cm^{-1} under the different chemical environments as explained in the main text (Fig. 5).

The six Raman modes (blue, yellow, and red lines) are the same modes as shown in Fig. 5. The gray line indicates the normal mode of the molecule in the gas phase. The image on the right shows detailed vibrational modes of the molecule in the gas phase.

Supplementary References

[1] Stadler, J., Schmid, T., & Zenobi, R. Nanoscale chemical imaging using top-illumination tip-enhanced Raman spectroscopy. *Nano Letters*, **10** (11), 4514-4520 (2010).

[2] Neacsu, C. C., Berweger, S., & Raschke, M. B. Tip-enhanced Raman imaging and nanospectroscopy: sensitivity, symmetry, and selection rules. *NanoBiotechnology*, **3** (3), 172-196 (2007).

[3] Stec, H. M., Williams, R. J., Jones, T. S., & Hatton, R. A. Ultrathin transparent Au electrodes for organic photovoltaics fabricated using a mixed mono-molecular nucleation layer. *Advanced Functional Materials*, **21** (9), 1709-1716 (2011).

[4] Maniyara, R. A., Rodrigo, D., Yu, R., Canet-Ferrer, J., Ghosh, D. S., Yongsunthon, R., Baker D. E., Rezikyan, A., García De Abajo, F. J., & Pruneri, V. Tunable plasmons in ultrathin metal films. *Nature Photonics*, **13** (5), 328-333 (2019).

[5] Mahmoodi, N., Rushdi, A. I., Bowen, J., Sabouri, A., Anthony, C. J., Mendes, P. M., & Preece, J. A. Room temperature thermally evaporated thin Au film on Si suitable for application of thiol self-assembled monolayers in micro/nano-electro-mechanical-systems sensors. *Journal of Vacuum Science & Technology A: Vacuum, Surfaces, and Films*, **35** (4), 041514 (2017).

[6] Jarrett, D. N., & Ward, L. Optical properties of discontinuous gold films. *Journal of Physics D: Applied Physics*, **9** (10), 1515 (1976).

[7] Lee, G. J., Kim, J. J., Hwangbo, C. K., Kim, J., Park, I., & Lee, Y. P. Optical properties of Ag hemisphere-like nanoparticles. *Journal of Nanoscience and Nanotechnology*, **13** (1), 568-571 (2013).

[8] Chizhik, A. I., Chizhik, A. M., Huss, A., Jäger, R., & Meixner, A. J. Nanoscale probing of dielectric interfaces with single-molecule excitation patterns and radially polarized illumination. *The Journal of Physical Chemistry Letters*, **2** (17), 2152-2157 (2011).

[9] Khoptyar, D., Gutbrod, R., Chizhik, A., Enderlein, J., Schleifenbaum, F., Steiner, M., & Meixner, A. J. Tight focusing of laser beams in a λ/2-microcavity. *Optics Express*, **16** (13), 9907-9917 (2008).
